# Supplementary material for: The nanoscale organization of the Wnt signaling integrator Dishevelled in the vegetal cortex domain of an egg and early embryo
Source: PLoS One. 2021 May 26;16(5):e0248197. doi: 10.1371/journal.pone.0248197 (PMC8153439; doi:10.1371/journal.pone.0248197)
Supplement: S1 Table — (A) Shows density of Dsh puncta per square micron for LM regions of VCD (edge, midway and center) and TEM images used for graphs in Figs 1J and 3G. (B) One-way ANOVA analysis of Dsh puncta per square micron data for three LM regions of VCD shown in graph in Fig 1J. (C) Dsh aggregate area for TEM images of UF cortices from the two species which appears in graph in Fig 3H. (D) Results of t-test analysis of Dsh aggregate area data in panel C and Fig 3H. (E) Data for comparing Dsh puncta density in the central VCD in unfertilized eggs vs. first cleavage stage embryos shown in the graph in Fig 7F. (F) Results of t-test of analysis of Dsh punctate density data in panel E and Fig 7F. (PDF) [file pone.0248197.s001.pdf]

S1 Table– for Henson et al., 2021 submitted to *PLoS One*

LM Edge	LM Midway	LM Center	TEM
0.56	1.52	2.92	1.57
1.12	1.4	2.68	1.86
0.4	1.2	2.48	2.39
1.08	1.2	2.84	2.26
0.56	2.36	2.76	2.79
1.12	1.36	2.64	1.4
0.36	1.48	2.84	2.12
0.44	1.96	3.16	1.96
0.84	1.52	2.8	1.34
0.16	2.28	3.68	1.74
0.28	3.04	3.6	1.88
0.24	2.12	3.2	2.38
0.6	2.08	3.56	1.85
0.32	1.72	3.8	1.66
0.44	2.16	3.8	2.15
0.96	2.68	2.88	1.10
0.36	3	3.68	0.79
0.24	3.52	3.88	1.42
0.36	2.88	4.32	1.61
1.04	3.48	4.6	0.81
1.12	2.08	4.12	1.08
0.92	2.4	3.96	2.21
0.96	2.84	5.32	2.40
0.36	2.84	5.24	2.30
0.6	2.32	3.72	1.50
0.64	2.6	4.68	
1.12	1.4	4.6	
0.6	1.64	4.88	
0.68	2.04	4.96	
0.88	1.56	5.28	
0.96	1.56	2.4	
0.8	2	2.72	
0.68	1.8	2.44	
0.6	1.92	3	
0.72	1.44	2.52	
0.68	1.52	2.72	
0.64	1.24	3.04	
0.48	1.48	2.24	
0.32	1.28	2.44	
0.6	1.28	3.16	
0.88	1.2	3.32	
0.68	1.92	2.72	
0.68	1.88	3.16	
0.68	1.48	2.96	
0.44	1.64	3.24	
0.72	1.4	3.32	
0.4	1.16	3.04	
0.32	1.16	3.32	
0.24	1.04	4.04	
0.56	1.24	3.6	
0.56	1.6	4.64	
0.52	1.64	4.08	
0.96	2	4.68	
1.28	1.56	4.48	
1.08	1.76	4.6	
0.4	1.8	4.36	
0.44	1.44	3.92	
0.52	1.48	4.12	
1.16	1.36	4.64	
0.92	1.28	4.28	

PANEL A - for graphs in Figs 1J & 3G

Ordinary one-way ANOVA					
Table Analyzed	Data 1				
Data sets analyzed	A-C				
**ANOVA summary**					
F	345.4				
P value	<0.0001				
P value summary	****				
Significant diff. among means (P < 0.05)?	Yes				
R squared	0.7961				
**Brown-Forsythe test**					
F (DFn, DFd)	26.52 (2, 17				
P value	<0.0001				
P value summary	****				
Are SDs significantly different (P < 0.05)?	Yes				
**Bartlett's test**					
Bartlett's statistic (corrected)	58.41				
P value	<0.0001				
P value summary	****				
Are SDs significantly different (P < 0.05)?	Yes				
**ANOVA table**	**SS**	**DF**	**MS**	**F (DFn, DFd)**	**P value**
Treatment (between columns)	263.9	2	131.9	F (2, 177) = 345.4	P<0.0001
Residual (within columns)	67.60	177	0.3819		
Total	331.5	179			
**Data summary**					
Number of treatments (columns)	3				
Number of values (total)	180				

PANEL B - for graph in Fig 1J

L. pictus	*S. purpuratus*
15617	8609
13070	28784
11264	9447
10387	15980
15143	22949
12608	12902
9456	12567
14243	18579
14320	16437
22267	11634
14900	15195
17049	13335
24450	11348
19762	13833
16001	13955
12546	10684
19383	8350
20957	24894
32312	24528
20103	34168

PANEL C – for graph in Fig 3H

Unpaired t test	
Table Analyzed	Data 1
Column B	*S. purpuratus*
vs.	vs.
Column A	*L. pictus*
**Unpaired t test**	
P value	0.8496
P value summary	ns
Significantly different (P < 0.05)?	No
One- or two-tailed P value?	Two-tailed
t, df	t=0.1909, df=38
**How big is the difference?**	
Mean of column A	16792
Mean of column B	16409
Difference between means (B - A)	-383.0 ± 2006
95% confidence interval	-4444 to 3678
R squared (eta squared)	0.0009583
**F test to compare variances**	
F, DFn, Dfd	1.687, 19, 19
P value	0.2632
P value summary	ns
Significantly different (P < 0.05)?	No
**Data analyzed**	
Sample size, column A	20
Sample size, column B	20

PANEL D - for graph in Fig 3H

UF-VCD Center	CL-VCD Center
3.8	3
4.5	2.7
3.7	2.4
4.4	2.3
3.5	2
3.6	2.1
3	1.8
3	2.1
2.8	2
3.2	2.1
2.8	2.6
2.4	2.4
2.8	2.1
2.7	1.5
3	1.6
2.6	1.5
3.9	1.8
4.1	1.6
3.6	1.4
4.7	1.8

PANEL E - for graph in Fig 7F

Unpaired t test	
Table Analyzed	Data 1
Column B	CL-VCD Center
vs.	vs.
Column A	UF-VCD Center
**Unpaired t test**	
P value	<0.0001
P value summary	****
Significantly different (P < 0.05)?	Yes
One- or two-tailed P value?	Two-tailed
t, df	t=7.594, df=38
**How big is the difference?**	
Mean of column A	3.405
Mean of column B	2.040
Difference between means (B - A) ± SEM	-1.365 ± 0.1797
95% confidence interval	-1.729 to -1.001
R squared (eta squared)	0.6028
**F test to compare variances**	
F, DFn, Dfd	2.441, 19, 19
P value	0.0588
P value summary	ns
Significantly different (P < 0.05)?	No
**Data analyzed**	
Sample size, column A	20
Sample size, column B	20

PANEL F - for graph in Fig 7F
